# Gender Disparities in Paradoxical Hypertrichosis After Laser Hair Removal

**DOI:** 10.1111/jocd.70194

**Published:** 2025-04-29

**Authors:** Sho Moriguchi

**Affiliations:** ^1^ Crest Skin Clinic Chiba Japan; ^2^ Keio University School of Medicine Tokyo Japan

**Keywords:** GentleMax Pro, hair removal, hair removal laser, paradoxical hypertrichosis

Paradoxical hypertrichosis (PH) is a rare but significant side effect of laser hair removal (LHR), characterized by unexpected hair growth in treated areas. While previous studies have reported an overall incidence of approximately 0.6%–10%, the impact of gender on PH remains poorly understood [1, 2]. Given the predominance of female subjects in LHR research, sex‐based differences in PH risk have not been thoroughly evaluated. To investigate potential gender differences in PH incidence, we conducted a retrospective chart review of 318 patients (63 males, 255 females) treated at our clinic from March 2022 to January 2024. All patients received treatment on various body areas using two laser platforms: the GentleMax Pro and GentleMax Pro Plus (755 nm alexandrite/1064 nm Nd:YAG; Candela). Laser parameters (fluence, pulse width, spot size) were set according to each manufacturer's recommendations, with adjustments by experienced staff to optimize efficacy and safety. All patients had an East Asian skin phototype II–IV. Clinical evaluations were conducted by both trained nurses and physicians to confirm PH occurrence.

Our analysis revealed a significantly higher incidence of PH in males compared to females (33.3% vs. 9.0%, *p* < 0.05). Notably, the anatomical distribution of PH differed between genders. In males, the most affected areas were the back (10/63, 15.9%), upper arms (7/63, 11.1%), and shoulders (5/63, 7.9%). In females, PH was more frequently observed on the face (9/255, 3.5%), neck (8/255, 3.1%), and upper arms (5/255, 2.0%). Extensive PH affecting multiple areas was more prevalent in males (11/63, 17.4%) than in females (4/255, 1.6%).

The mechanisms underlying this gender disparity remain speculative but may involve differences in hair density, hormonal influences, and skin response to laser treatment. Androgen‐driven follicular activity in males, combined with the higher terminal hair density on the back and shoulders, may predispose men to PH when treated with suboptimal fluences. A recent study by Inoue et al. identified factors associated with PH, noting that daily sun protection significantly reduced its incidence, whereas no other specific preventive measures were reported [3]. Although the incidence rate of PH reported by Inoue et al. was lower than ours, their study included only female patients, which may explain the lower PH incidence. Additionally, it is not yet known whether lower or higher fluences are more likely to cause PH. In our study, all treatments followed manufacturer‐recommended fluences, yet PH still developed, emphasizing the need for further investigation into individualized laser fluences. Furthermore, our results suggest that PH is not confined to facial areas, as often reported, but can affect diverse body sites based on gender‐specific patterns. Practitioners should recognize the elevated PH risk in male patients, particularly on the back, shoulders, and upper arms. Optimizing laser parameters, using appropriate fluences for different anatomical regions, and considering alternative treatment strategies such as needle hair removal may help mitigate this risk.

If PH occurs, continuing treatment with adjusted parameters may be beneficial [2]. Some reports suggest that persistent laser exposure can eventually reduce paradoxically induced hair growth. Future studies should explore whether modifying fluence, pulse duration, or laser wavelength can prevent or reverse PH in high‐risk populations.

In conclusion, our study demonstrates a significant gender difference in PH incidence following LHR, with males exhibiting a markedly higher risk. These results highlight the importance of gender‐specific considerations in LHR protocols and warrant further research to refine treatment approaches for improved patient outcomes.

## Conflicts of Interest

The author declares no conflicts of interest.

## Data Availability

The author has nothing to report.
